# Community perspectives on barriers to injury care in Northern Malawi: a three delays framed assessment using focus groups and photovoice

**DOI:** 10.1186/s12913-024-11890-4

**Published:** 2024-11-12

**Authors:** John Whitaker, Ella Togun, Levie Gondwe, Donaria Zgambo, Abena S. Amoah, Albert Dube, Rory Rickard, Andrew JM Leather, Justine Davies

**Affiliations:** 1 Institute of Applied Health Research, University of Birmingham, Birmingham, UK; 2 School of Life Course and Population Sciences, King’s College London, London, UK; 3 Academic Department of Military Surgery and Trauma, Royal Centre for Defence Medicine, Birmingham, UK; 4 Malawi Epidemiology and Intervention Research Unit (formerly Karonga Prevention Study), Chilumba, Malawi; 5 Faculty of Epidemiology and Population Health, London School of Hygiene & Tropical Medicine, Keppel Street, London, UK; 6 Department of Parasitology, Leiden University Center for Infectious Diseases, Leiden University Medical Center, Leiden, the Netherlands; 7 Medical Research Council/Wits University Rural Public Health and Health Transitions Research Unit, Faculty of Health Sciences, School of Public Health, University of the Witwatersrand, Johannesburg, South Africa; 8 Department of Global Surgery, Stellenbosch University, Stellenbosch, South Africa

**Keywords:** Photovoice, Focus group discussion, Health system research, Low- and middle-income countries, Injury, Trauma

## Abstract

**Introduction:**

The global burden of injury is huge, falling disproportionately on poorer populations. The benefits of qualitative research in injury care are recognised and its application is growing. We used a novel application of focus group discussions with photovoice to rapidly assess barriers at each of three delay stages; seeking (delay-1), reaching (delay-2) or receiving (delay-3) injury care in Northern Malawi.

**Methods:**

Three community Focus Group Discussions (FGDs) of individuals with (FGD1) and without (FGD2) recent injury experience and community leaders (FGD3) discussed barriers to seeking, reaching or receiving care following injury. Participants from FGD1 subsequently used a digital camera and, following training in photovoice, took photographs illustrating barriers to injury care. Participants reconvened to discuss images which they believed illustrated important barriers. A framework method analysis compared barriers generated to those identified by an earlier Delphi study.

**Results:**

Seven of eight invited adult community members attended each discussion group. Within the FGDs, all prior Delphi derived delay 1 barriers were described. Within delay 2, all but three were discussed by community participants. Those not covered were: 1) “*communication*” ;2) “*prehospital care*”; 3) “*coordination*”. Within Delay 3, only “*capacity*”was not highlighted by participants during the study.

Additional health system barriers not identified in the Delphi were inductively derived. Within Delay 1, these were labelled; “*religious or other beliefs*”; “*indecision*”; “*fear or lacking courage*”; and “*community/bystander engagement*”. Within Delay 2, “*lack of assistance*” was derived. Within Delay 3; “*alleged corruption*”; “*interfacility transfer*”; and “*police processes*” were all identified during analysis.

The photovoice group provided 21 photographs evidencing 15 barriers. Delay 1 was the most frequently captured by images (12/21).The individual barriers most frequently described were “*transport*” and “*roads*” (6/21 and 5/21, respectively). The photovoice group did not describe any additional barriers not covered in the prior FGDs.

**Conclusion:**

We identified several barriers within this health system. Participants illustrated how some barriers impact on multiple phases of delay. The method was quick, low cost and participants grasped the technique and research question effectively. We recommend this approach for future health system assessments.

## Background

Each year injuries cause 6 million deaths worldwide [1] and disproportionately affects poorer populations with 90% of injuries occurring in low- and middle-income countries (LMICs). Africa in particular bears a substantial proportion of the 63 million global injury Disability Adjusted Life Years (DALYs) [1, 2]. 

In Malawi 6.4% of deaths are linked to injury-related causes [3]. Injuries are a common cause of facility attendance and avoidable preventable death predominantly affecting young males [1, 2, 4, 5]. Personal experience of, and insight into, injury and injury care are common, 40% of schoolchildren having witnessed a person being hit by a motor vehicle [6]. Obtaining reliable data on injuries is a challenge. The content of routinely collected medical records in Malawi is not of sufficient quality to serve this purpose [2, 3]. The little trauma registry data available cannot evaluate aspects such as health-seeking behaviour for which little published literature is available [7, 8].

The Lancet Commission on High-Quality Health Systems in the Sustainable Development Goal era advocates for increased involvement of people and communities in research, to deepen understanding of health systems [9]. Health systems that provide quality care are people-centred; necessitating community engagement in planning. Information obtained from communities provides context-relevant information to identify system gaps, explain health seeking behaviours, monitor performance and identify the optimal ways to use finite resources [9, 10]. 

Health system barriers can hinder timely access to the quality of care that injured patients need and may prevent them receiving any care at all [11]. The Three Delays framework has supported better understanding of the determinants of maternal mortality, and has been used to evaluate health systems and facilitate quality improvement more broadly [12, 13]. This framework considers barriers to seeking (delay one), reaching (delay two) and receiving (delay three) care [14]. It has been advocated for evaluating urgent care, including injuries, in LMICs [11, 15, 16]. We previously used a Delphi process to derive expert consensus on important barriers to evaluate to understand a health system’s ability to care for the injured. We identified 20 conceptual barriers which were categorised within a Three Delays framework to guide future health system evaluation and strengthening in LMIC settings [17]. 

The benefits of qualitative research in injury care are recognised and its application is growing [18, 19]. Focus group discussions (FGDs) generate data through communication between participants [20]. They allow examination of lived experience of disease and care services [21]. Acknowledged as accessible and convenient, FGDs can quickly generate large amounts of data [22]. Participants are encouraged to discuss issues, share anecdotes, points of view and experiences [23]. They can be useful when exploring cultural norms, areas of criticism and embarrassing subjects [24]. They allow those unable to read or write to participate along with those intimidated by formal interviews [24]. Adding visual and physical tools such as cards or “spidergrams” can help stimulate discussion, particularly to explore consensus [24–27]. FGDs have been used to explore community perceptions of emergency and trauma care systems in other LMICs to provide data on community members’ experience of barriers to care [28–30]. They are well suited to exploring complex topics to understand what a community believe about a system and why [22]. 

Photovoice is a relatively new research methodology conceived in the 1990s with the original aims of reflecting community concerns, facilitating critical group discussion around important subjects revealed through photographs and influencing policymakers [31]. It has gained popularity within global health research and has been applied within Malawi to study relationships between cooking and pneumonia [32], resilience amongst the poor [33], activities amongst adolescents [34] and the contribution of palliative care in advanced cancer [35]. The use of photovoice in injury research is in its infancy, focused on either prevention or rehabilitation and set in high-income countries (HIC). In HIC settings it has been used to explore children’s experiences of forearm fracture and lessons for prevention [36], for reducing pedestrian injuries in children [37], and the experiences, barriers and facilitators to social reintegration following spinal cord injury [38–42] and burns [43]. Using photovoice for LMIC injury care health system research is therefore novel. Using both FGDs and photovoice to explore barriers to injury care within a Three Delays framework represents an innovative approach to obtain community perspectives on factors acting as health system barriers.

This study aimed to establish community perspectives on factors acting as barriers or facilitators to care following injury within a Three Delays framework through Focus Group Discussion and photovoice methods.

## Methods

### Study setting

Malawi is a low-income country (LIC) in south-eastern Africa with a population of 19 million, most of whom dwell rurally and live in poverty [2, 44]. The study was conducted at the Karonga Health and Demographic Surveillance Site (HDSS) of the Malawi Epidemiology and Intervention Research Unit. It has an established history of community research with strong relationships with the community traditional authorities [45]. The unit also has existing research infrastructure including trained research assistants practised in facilitating qualitative methods [46, 47] such as focus groups. The HDSS has a population of over 40,000 under surveillance [45]. It is based in the surrounds of Chilumba, in the south of Karonga district, typical of a Malawian subsistence economy reliant on farming and fishing [45]. The climate is hot and dry from September to December, rains from January to May, and is cool and dry from June to August.

The HDSS population are mostly rural, although approximately 15% live in semi-urban settlements of a trading centre and the portside village of Chilumba. Half of the population live within one kilometre of a tarmac road [45]. The main tarmac road runs through the district with mostly gravel secondary roads. The local vernacular language of Chitumbuka is spoken throughout Karonga and within the population of the HDSS.

Government facility healthcare is free at point of use to all residents of Malawi, although out-of-pocket payments, such as patient and relative transport and sustenance costs, can occur [48]. Whilst traditional healers have recognition as deliverers of health services, links with the formal health system are sparse [49]. The private sector includes private for-profit and private not-for-profit providers. In particular the Christian Health Association of Malawi (CHAM) provides healthcare worker training and provides services [50]. The government purchase services from CHAM, particularly essential health services for populations remote from public facilities [51]. Malawi’s three-tiered system includes primary health services; these consist of community initiatives, health posts, dispensaries, maternity units, health centres and community and rural hospitals [50]. District hospitals constitute secondary healthcare and provide services to patients referred from primary care. Tertiary healthcare, for specialised services, is provided by central hospitals. Malawi has only 0.036 physicians per 1000 population, one of the highest ratios of non-physician clinicians to population and most clinicians are non-physicians [52]. No established prehospital emergency medical service exists. Injured patients frequently attend primary facilities at first rather than bypassing directly to secondary or tertiary care.

### Data collection

#### Participant selection

Three community Focus Group Discussions (FGDs) were undertaken. Eight adults (> 18 years) were purposively selected for each. The first consisted of members of the general public who had recently (within the past 12 months) sustained an injury. The injury had to have been severe enough to inhibit usual activities for at least one day, or resulted in accessing formal injury care, or both. These participants were identified through the Karonga HDSS key informant network embedded within the local community [53]. The second consisted of adult members of the general public without requiring recent experience of significant injury to be included. They were identified from households close to those of the first focus group participants. The third consisted of community leaders identified through the traditional authority network within the HDSS. Eligible participants were purposively invited to include a mix of mechanism of injury (for the first FGD), gender, age, and household location. Limiting to 3 FGDs was an a priori decision based on available resources and permissions obtained.

#### Participant recruitment

Two native Chitumbuka speaking research assistants (RAs) visited these individuals in their communities to explain the project using the participant information sheet at least 24 h in advance of the discussion group meetings. These same RAs assisted in conducting the group discussion.

#### Discussion group conduct

After a period of training, a RA facilitated the FGDs in Chitumbuka, using a translated discussion guide. For participant convenience, FGDs were conducted at a centrally positioned building in the HDSS. Two employed fluent Chitumbuka speaking RAs (one male, one female) with prior experience conducting qualitative research within the local population and two project researchers (one male, one female) facilitated the discussions. Participants were not known to the research team prior to the study.

To help orientate participants to the subject of injuries, the RAs provided examples of physical injuries using four hypothetical injured patients encompassing a range of injury mechanisms and social contexts [54]. These were (1) a farmer kicked in the chest by a cow sustaining a blunt chest injury causing tension pneumothorax, (2) a young adult male stabbed during an assault outside a bar causing penetrating abdominal injury with hypovolaemic shock, (3) a young adult female falling from the roof of her home sustaining a severe head injury, and (4) a motorcyclist hit by a taxi on the main road and sustaining an isolated lower-limb open fracture. Then they asked participants to describe, in order, potential barriers then facilitators to care-seeking (Delay 1), reaching care (Delay 2) or receiving quality care (Delay 3) following injury within their community. To aid discussion “spidergrams” were created upon which the barriers and facilitators were placed. ‘Spidergrams’ are visual tools for identifying and analysing relationships. The spider’s body may be used to define the focus (in this case a conceptual delay) with the legs used to reflect factors relevant (in this case barriers or facilitators) [27]. Discussions lasted approximately 90 minutes. FGDs were audio-recorded and subsequently transcribed and translated into English by Malawi Epidemiology and Intervention Research Unit (MEIRU) trained staff. The “spidergrams” were photographed for use in analysis as field notes. Discussions continued until no additional factors acting as either a barrier or a facilitator to injury care were proposed; this was deemed to represent conceptual saturation for each discussion group. Discussions were constructed to explore both barriers and facilitators to encourage capturing as many important potential health system barriers as possible.

Participants from the first FGD (injured in the past 12 months) were then invited to continue as participants in the photovoice study. There were otherwise no repeated interviews or discussions involving the other FGD participants. All participants from the first FGD all agreed to continue as participants in the photovoice study. The photovoice study is conceived as an extension of the community FGD study.

**Fig. 1:**
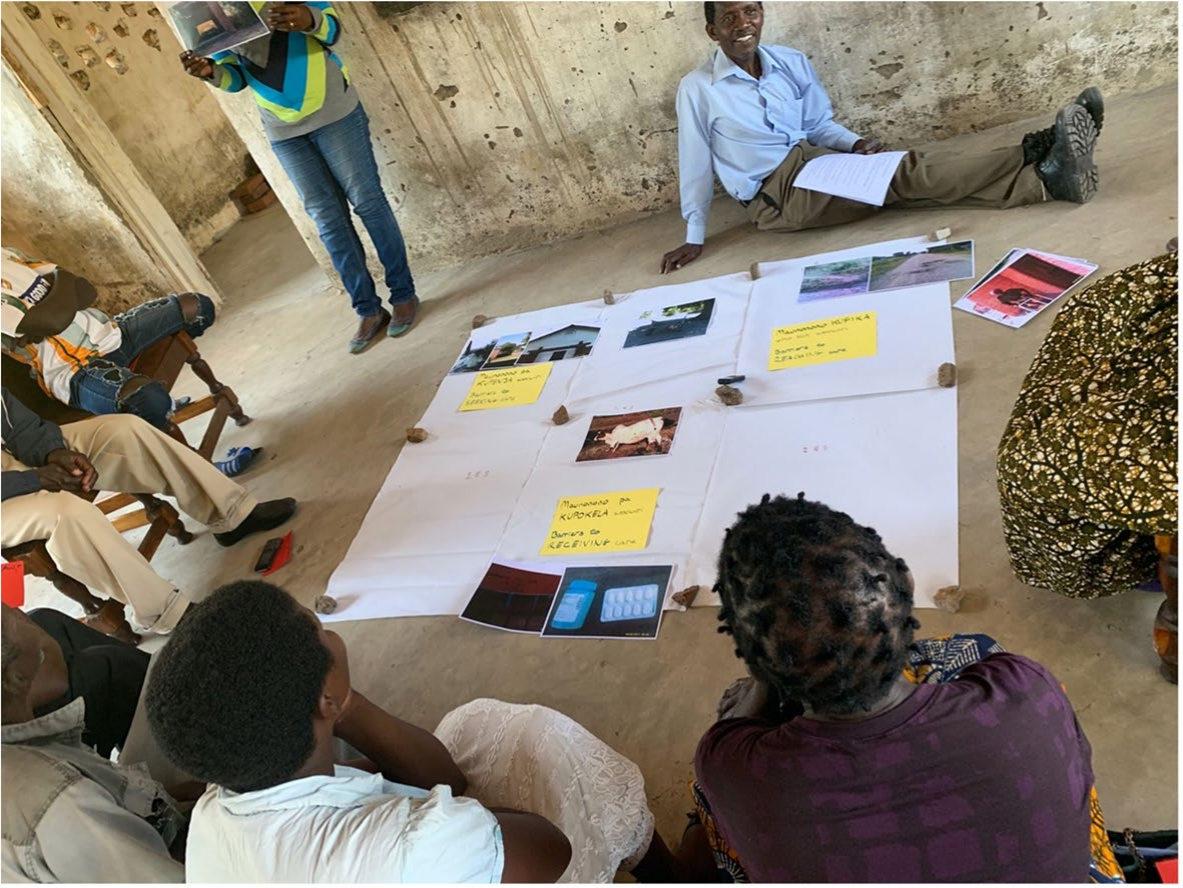
Conduct of Photovoice workshop discussion showing pictures being placed in position corresponding to the relevant delay(s) described (consent obtained for use of participant images)

Photovoice participants received a basic digital camera to use. They returned the camera to the research team at the end of data collection. A training session was delivered on using the camera, fundamentals of photography, ethical considerations when photographing people, photovoice principles, and the study objectives [55]. Participants were instructed to use photography to visually depict barriers they perceived as important to seeking, reaching and receiving high quality healthcare after injury. Participants had a hard copy training manual to refer to and allowed one week to capture the photographic images. The research team visited the home of participants to clarify and resolve any problems and ensure satisfactory progress. Participants were again visited at the end of the week and asked to choose three preferred images that illustrated the barriers they wanted to highlight and gave labels for the photos. The photos were then printed on A4 laminate paper. The following day, all photovoice participants met together at the original location for a further discussion. Participants were asked to elucidate the significance of each photo and why they chose to highlight it. As part of this discussion participants collectively mapped each image and the barrier(s) represented to the Three Delays framework. Images were positioned on a Venn Diagram framework positioning each delay or overlapping delays (Fig. 1). Participants also shared their experiences of taking part in the photovoice study. This included any difficulties taking photos they would ideally have captured but were unable to. The discussion was recorded. Where recognisable people were included in the photos they were located and their express written consent was sought for use of their image. In case individual could not be found, then the photos were to be blurred so individuals could not be recognised. Audio recordings were transcribed and translated into English by trained native Chitumbuka speakers. The transcriptions could not be confirmed with participants for logistical reasons.

#### Analysis

Analysis of both the FGD and photovoice study data was conducted concurrently. Data analysis was conducted using the framework analytical method, including familiarisation; identifying a thematic framework; indexing; charting; and interpretation [56, 57]. This method is well matched to this study’s specific research question. It is suitable when time frames are limited, participant samples are clearly defined, and the issues of focus have been considered a priori. Although framework analysis may generate novel theories, the prime objective is to describe and deduce what is happening in a particular setting. For the purpose of this study analysis the a priori framework was the Three Delays framed barriers to injury care generated from a prior Delphi expert consensus study [17]. This Delphi derived framework identified barriers important to evaluate to understand a health system’s ability to care for the injured. It identified 7 barriers to seeking care (cost, perceived physical access, perceived care quality, delayed discovery, traditional healers, healthcare literacy, cultural norms), 6 barriers to reaching care (communication, transport, pre-hospital care, distance, coordination, roads), and 7 barriers to receiving care (staff, specialists, physical resources, patient cooperation, quality processes, payment, capacity). This framework was used as a starting point for deductive analysis with scope for additional barriers and themes emerging through inductive analysis. Data was coded using NVivo 12 software [58]. Discussion transcripts, photovoice images and “spidergrams” were all used to aid interpretation. Barrier themes additional to those from the Delphi study were generated. For this analysis, barriers and facilitators were consolidated to allow interpretation of reciprocal descriptions, i.e. a lack of something can be a barrier, but a presence of the same can be framed as a facilitator [59–61]. 

#### Ethical considerations

A community sensitization meeting took place one week before the first FGD at a convenient central location within the HDSS. Traditional heads were invited to attend a meeting where all aspects of the community studies were explained, and questions answered. This is routine practice on the introduction of new studies within the MEIRU Karonga HDSS [45]. The study was explained to all study participants. They were provided with a participant information leaflet and consent form which they read or was read out to them in Chitumbuka by a native speaking research assistant. Any questions about the study conduct were answered, and participants signed or provided a thumbprint on a consent form, confirming their agreement to participate in the study. This was witnessed. The study was approved by the Malawi National Health Sciences Research Committee (ref 19/03/2263) and the UK MOD Research and Ethics Committee (ref 960/MODEC/19). Where identifiable images are used, participant consent was obtained.

## Results

Out of eight invited individuals, seven attended each discussion group (all 3 FGDs and photovoice). The reasons for non-participation were not established. The demographic characteristics of participants are shown in Table 1. Each FGD meeting took between 70 and 100 min.

**Table 1:** Community FGD and photovoice participant characteristics. FGD – Focus Group Discussion

Injured community member and photovoice participants	
Age	Median 46 Range 20–74
Sex	3 Females, 4 Males
Occupation	6 farmers, 1 fisherman
Injury Mechanism	3 Road Traffic, 2 Fall, 1 struck or hit by a person, 1 fire, flames or heat
Injured body part	4 head, 3 arms, 3 legs, 1 hand (more than one body part possible)
Injury Type	4 experienced open wounds, 3 internal injuries, 2 broken bones, 1 burn. (NB more than one injury type possible per person).
Days unable to perform usual activities	Median = 60 Range 30–120 days
Time since injury (months)	Median 7, range 2–11
Marital status	5 married, 2 other
Unable to perform usual activities currently	4 reported still being unable to perform usual activities
Received healthcare after the injury?	All received healthcare following injury
Uninjured community member participants	
Age	Median 33 Range 29–51
Sex	6 female 1 male
Occupation	6 famers 1 businessperson
Marital status	5 Married 2 other
Community leader participants	
Age	Median 47 Range 37–60
Sex	4 Female 3 Male
Occupation	6 Farmers and 1 Health Worker
Marital status	4 Married 3 Other

Within the FGDs, all Delphi derived Delay 1 barriers were described (Table 2). Within Delay 2, all but three Delphi derived barriers were discussed by community participants. Those not covered were: (1) “*communication*” - there is a lack of an accessible emergency assistance communication mechanism (e.g. emergency call centre); (2) “*prehospital care*” - there is a lack of timely available prehospital emergency care (formal or informal/bystander); (3) “*coordination*” - There is a lack of emergency care service coordination, including bypassing unsuitable facilities or transferring between facilities. Within Delay 3, only the Delphi derived barrier of “*capacity*” - in regard to patient demand, there is insufficient facility capacity to meet patient demand (e.g. overcrowding), was not highlighted by participants during the study.

**Table 2:** Barriers identified and number of FGDs and photos describing them in community FGDs and photovoice studies. FGD 1 was injured community members, FGD 2 was uninjured community member participants, FGD 3 was community leader participants. FGD – Focus Group Discussion

Barrier	Summary of themes raised	FGDs describing barrier (*N*)	Photos describing barrier (*N*)
Delay 1			
Cost	Payments required to access care including: • Direct transport costs • Indirect costs of care such as food • Leaving uninjured family members at home without sufficient financial resources • Need to earn and preserve financial resources for emergency.	3	3
Perceived physical access	The physical distance between place of injury and a care facility. A shorter distance to care lessens the financial burden associated with transport. Topography can put off travel Rainy season is an exacerbating factor	2 (FGD 2&3)	0
Perceived care quality	Past experience can drive this perception. Non-technical aspects including clinician attitudes emphasised. Structural components of care quality, such as perceived lack of physical resources included. Positive experience and outcome could incentivise care-seeking.	3	1
Traditional healers	Preference for traditional healers as providers of care framed universally as negative. Derives from an underlying belief structure that witchcraft can cause accidental injuries. Serious injuries using traditional healing methods seen as potentially dangerous.	3	3
Religious or other beliefs^a^	Beliefs in healing through prayer from churches reported to cause delay in health-seeking.	1 (FGD 1)	2
Healthcare literacy	Perceived injury severity influenced health-seeking behaviour. Both sight of blood and experience of severe pain likely to increase perception of severity. Severe injuries can go unrecognised by both the injured person and community members. Community members lack awareness in injury response prompting calls for further education and legislation. Perceived futility in high severity injuries.	3	0
Cultural norms	Male household heads traditionally make health-seeking decisions for the family. Financial burden to seeking care may inhibit financing other family necessities. Family or neighbours may be too preoccupied with their own activities to assist.	1 (FGD 3)	0
Delayed discovery	A child’s injury might not be reported to a parent	1 (FGD2)	0
Indecision^a^	Patient or carer “dillydallying” could cause delay.	3	1
Fear or lacking courage^a^	Being emotionally incapacitated by an unwell family member or child.	2 (FGDs 2&3)	0
Community or bystander engagement^a^	Bystanders are key care-seeking enablers and need courage to assist. Bystanders’ disagreements and lack of unity of action can cause delay. Communities should be engaged in caring for each other. Community leadership could help improve healthcare quality. Local bylaws could require prompt health-seeking following injury.	2 (FGDs 2&3)	3
Delay 2			
Communication	Not discussed.	0	0
Transport	There is a need to readily locate means of transport. The cost of transport is problematic and dependent upon various means. These include: Human beings / carrying patient, wheelbarrow, oxcart, bicycle, motorcycle, cars / motor vehicle. Cars are necessary for more severe injuries.	3	5
Prehospital care	Not discussed.	0	0
Distance	A large physical distance will delay reaching care itself. A large physical distance will restrict viable transport options.	2 (FGDs 2&3)	0
Coordination	Not discussed.	0	0
Roads	Unreliable condition delays reaching care. Marked deterioration in rainy season exacerbates this problem.	2 FGDs 1&3	6
Lack of assistance^a^	The community depend on others to help access care.	2 (FGDs 2&3)	0
Delay 3			
Staff	Staff may not be readily available especially outside routine working hours. Non-technical aspects of care highlighted include staff motivation and attitude.	3	1
Specialists	A lack of expertise to manage certain injuries locally deemed problematic as necessitates onward referral and contributed to avoidable morbidity and mortality. Visiting specialists at referral hospitals believed to promote accountability. Specialists could also motivate other clinicians to provide better care.	3	1
Physical resources	These is a lack of available medication, physical equipment and reliable infrastructure such as electricity.	3	2
Patient cooperation	Providing accurate information about the patient is necessary for good care as is compliance with the prescribed treatment Guardians advocating for patients should be aware clinicians have other patients to care for.	2 (FGDs 1&3)	0
Quality processes	Out of hours care arrangements involve inherent delays. Care must prioritise urgent injured cases based on clinical need.	2 (FGDs 1&3)	1
Payment	Direct care payments are required at private, including faith based, providers. Patients perceived to be wealthy could receive prompt and better care.	2 (FGDs 1&2)	2
Capacity	Not discussed	0	0
Alleged corruption^a^	Additional payments are allegedly sometimes required to receive care promptly, although this is no guarantee. Being well known to facility staff could facilitate prompt treatment.	2 (FGDs 1 &2)	2
Interfacility transfer^a^	Limited formal ambulance interfacility transfer is available. Alternative private transport or financing cost of fuel for ambulances often required.	2 (FGDs 1&3)	1
Police processes^a^	Injured persons can be required to obtain a police report prior to receiving care. This can be dependent on the mechanism of injury, especially those involving a third party. Facility staff can enforce this barrier.	1 (FGD 3)	0

^a^Barrier derived inductively

Additional health system barriers not identified in the Delphi [17] were inductively derived. Within Delay 1, these were labelled; “*religious or other beliefs*” – believing that seeking formal healthcare is itself wrong; “*indecision*” - relating to patient or carer delay through indecision; “*fear or lacking courage*” - irrational incapacitation or rational concern of consequences; and “*community/bystander engagement*” - not enough is done by fellow citizens to support care-seeking. Within Delay 2, “*lack of assistance*” - interdependence of community members for accessing care, was derived. Within Delay 3; “*alleged corruption*” - need for unauthorised payments or gifts to healthcare staff to receive best available treatment (e.g. corruption); “*interfacility transfer*” - lack of available means to safely and quickly transfer injured patients on to a more specialist facility; and “*police processes*” – perceived or actual police functions delay receiving care (such as the perceived need to be seen first by police following violence or road traffic collision), were all identified during analysis.

**Fig. 2:**
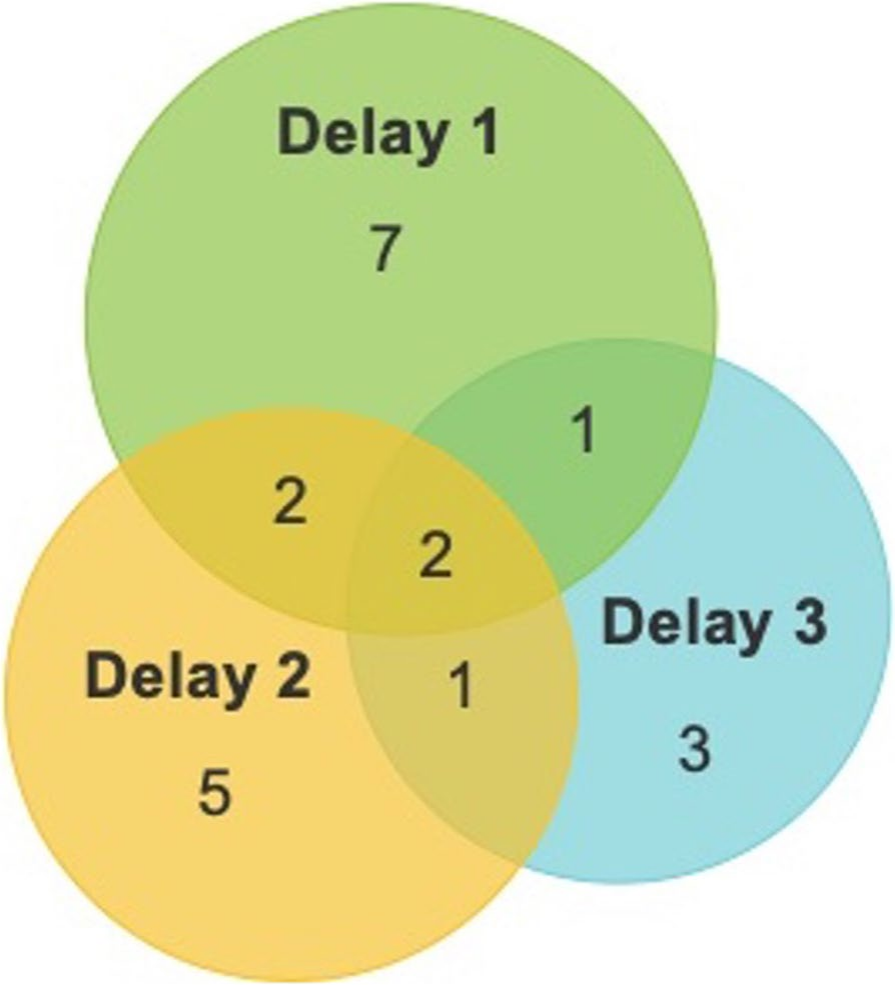
Number of photovoice study photographs mapped to each conceptual delay. Each circle of the Venn diagram represents a Delay. The number of photos mapped to each Delay alone or in combination is shown

The photovoice study provided photographs that evidenced 15 barriers across the Three Delays framework. Delay 1 was the most frequently captured delay by photovoice images (12/21, Fig. 2). The individual barriers most frequently described were “*transport*” and “*roads*” (6/21 and 5/21, respectively). Only one barrier evidenced through photovoice, “*community or bystander engagement”*, was not described in the preceding FGD with these same individuals (recently injured community member). However, both of the other FGDs did evidence this barrier, meaning the photovoice study did not describe any additional barriers to those identified in FGDs.

Each barrier identified by at least 2 FGDs and 2 photovoice images is now described in turn. They are structured within each conceptual delay with supporting participant quotations and photographs. Quotations are labelled according to the originating discussion group, “recently injured community member”, “uninjured community member”, “community leader”, and “photovoice”.

### Delay 1 seeking care

#### Cost – The financial costs associated with seeking care are too great

All three participant groups reported availability of financial means as a key determinant of healthcare access and the decision to seek care. Concern about payments required to access care, related to both direct transport costs and the indirect costs of care, including food, were highlighted. So too was concern about leaving uninjured family members at home without sufficient financial resources for daily life:

*“Sometimes transport is there … but you start thinking about what you’re going to eat at the hospital since there is no money to buy food and this may delay you .”* recently injured community member.

**Fig. 3:**
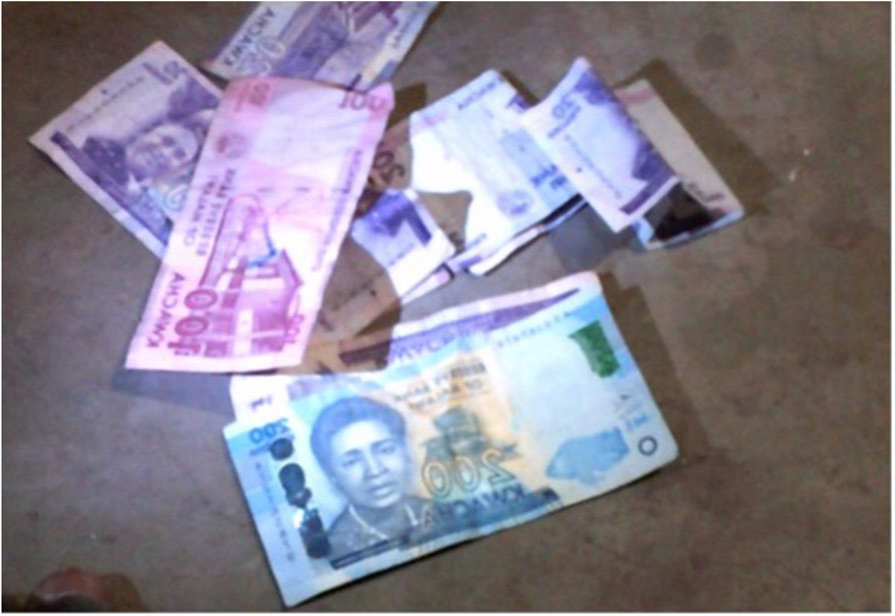
Kusowa kwa ndalama (lack of transport cost)

Photovoice participants also wanted to illustrate the importance of money, with two participants photographing notes of Malawian Kwacha:

– *“. when I had my own injury…there was totally nothing in my house. Fortunately*,* my neighbour assisted me; she gave me money and told me that I can pay her back when I am ready. And I used that money without which I will not have had any medical attention.” -* Photovoice participant with related photo in Fig. 3.

Participants proposed that community members should be prepared by earning and preserving financial resources to be able to access in an emergency. This idea was expressed through photovoice with livestock representing an asset to release financial means, or in the case of an ox and cart, a potential form of transport itself, juxtaposed to a tarmacked road:

*“people in the community have to be prepared by being active to fetch and keep money so that when (a) problem comes they should be quick to find transport to the hospital.” -* Uninjured community member.

**Fig. 4:**
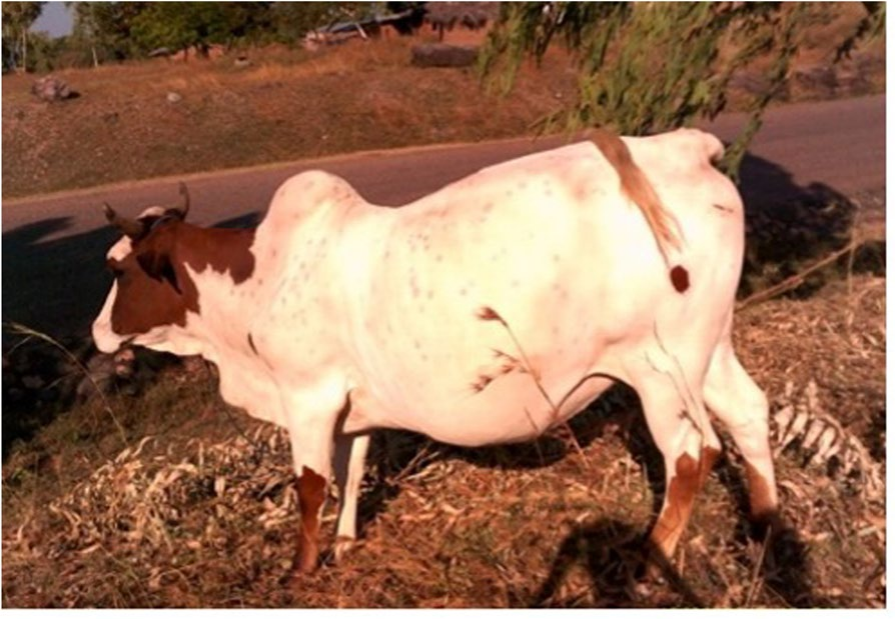
Nthowa ya kasangiro ka mendero (Means of finding transport or its cost). This photo illustrates several barriers

*“…*,* when I fell I did not have any money on me so I had to get one of my animals and I sold it so that I could be taken to the hospital.” -* Photovoice participant with related photo in Fig. 4.

#### Traditional Healers - People prefer traditional healers

The role of community member belief systems was strongly emphasised across the FGD participants. Participants highlighted that traditional healers acted as an important health system barrier to prompt care, expressing negative views about them, referring to witchdoctors as “liars”. Related to this was the reported worldview that witchcraft may have been responsible for accidental injuries in the first place:

*“If you had an argument with someone at some point people will attribute the accident to that argument. If you go to these liars (referring to witchdoctors) they will make you believe that you have been bewitched” -* Community leader.

*“At times we have made decisions to go to the hospital but because of the belief that we have we feel it is important to confirm that the accident was really natural by visiting the witchdoctors first and this causes a lot of delay.” -* Community leader.

**Fig. 5:**
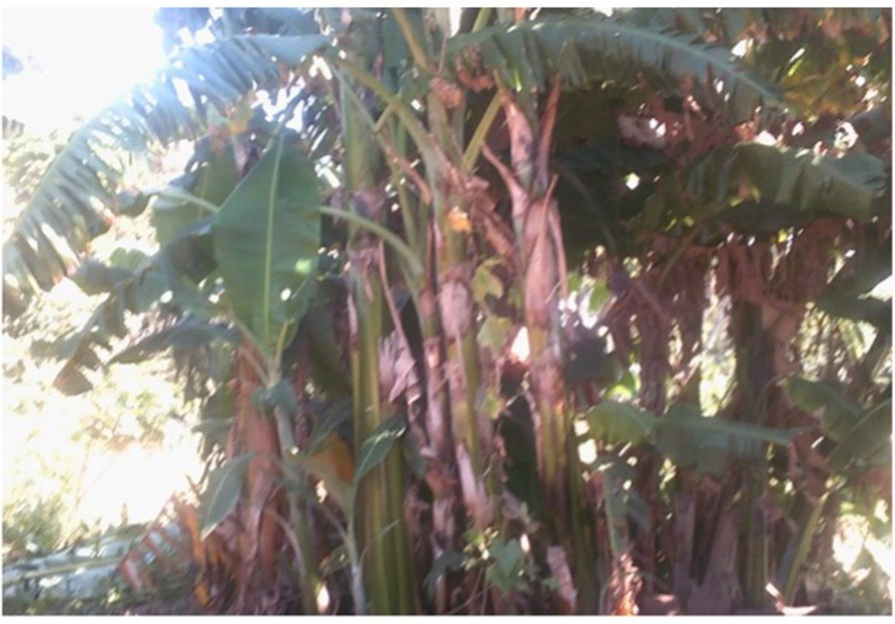
Musisi wa makombwe (roots of the banana tree)

Examples of serious injuries and violent mechanisms were illustrated from personal experience, framing reliance on traditional healing methods as potentially dangerous:

*“a cousin of mine got injured while playing football and he had a broken leg …when he got home my uncle wanted to take the boy to the hospital but the boy’s mother said no this is a simple issue we can treat it at home and then they took the banana roots*,* I don’t know what they added it with but that’s what they used to massage his leg. After one week he started swelling and it appeared …. like it was rotten. So*,* the lesson here is that we should go to the hospital first and we shouldn’t rely on traditional herbs” -* Photovoice participant with related photo in Fig. 5.

#### Community/bystander engagement - Not enough is done by fellow citizens to support care-seeking

Participants expressed that bystanders present at the time of injury could be key enablers of care-seeking. However, those bystanders also needed courage to assist.

*“I think the presence of courage is also important because if we have courageous people in the community and (if) an accident happens they can easily take the injured person to the hospital.” –* Community leader.

Similarly, the possibility of bystanders arguing over the best course of action can delay care-seeking, expressed as disagreement and a lack of unity of action:

*“The other thing that can delay a person to go to hospital is disagreements*,* sometimes when the person is severe injured instead of thinking to rushing with the patient to the hospital you delay because of disagreement.” -* Uninjured community member.

The value of a community that was engaged in caring for each other was expressed. Community leadership was mentioned with one photovoice participant photographing the meeting place for traditional authorities to discuss improving healthcare quality. Similar ideas were expressed for having local bylaws to require prompt health-seeking following injury:

*“Availability of bylaws these would help the community to go to the hospital as quickly as possible regardless of the size of the injury.” -* Community leader.

### Delay 2 reaching care

#### Transport - There is a lack of timely affordable emergency transport (formal or informal)

This barrier was repeatedly emphasised by all discussion groups, both in terms of whether or not a means of transport could be readily located or obtained and the cost of such transport. Motorised transport was described as necessary for injuries requiring attention at a referral facility. The patient condition and injury severity also influenced the transport choice. Transport options were broad and varied:

*“By transport here*,* I am not only referring to money because transport can mean anything … cars even human beings they can be referred to as transport.” -* Community leader.

*“transport has included a lot of means*,* for example a car which can help you to get to hospital very quickly and a motorcycle too*,* we also have bicycle which can also get you to the hospital though not very quickly and finally we have oxcart which can also help you get hospital while moving very slowly.” -* Recently injured community member.

*“. what will determine the type of transport …. is . being rich in the pocket …. you can have a wheelbarrow . and use it to take the patient to the hospital if it’s closer to the incident place and depending on the weight of the patient*,* … you can carry the patient on the back … according to severity of the injury …. and if it’s closer to the hospital. And maybe the good Samaritans on the way can come in to help take the patient to the hospital … and maybe if the bicycle is available*,* the patient can be taken to the hospital using the bicycle according to the condition of the patient …” -* Recently injured community member.

Motorcycles and bicycles could be important means of transport. However, a car, although more expensive, was required to reach more distant facilities:

*“Presence of (a) motorcycle or bicycle at the accident place can also help to carry the person to the hospital quickly.” -* uninjured community member:

*“even if the road is bad but the car is there it will surely get to the hospital … but even if the road is good and there’s no vehicle around*,* there is no way that you can get to the hospital.” -* Recently injured community member.

**Fig. 6:**
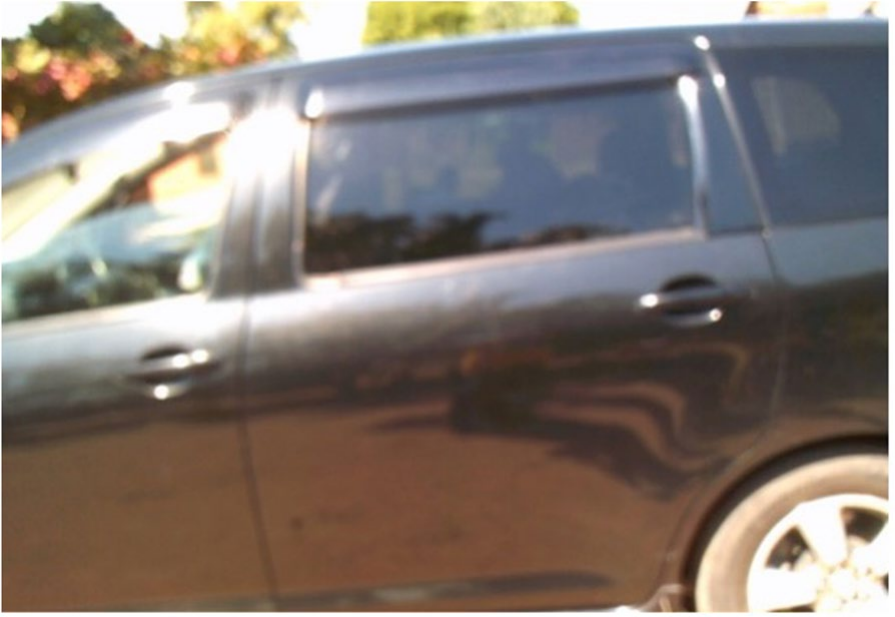
Mendero ya luwiro (quick transportation)

*“I took a picture of a car. The decision has already been made that the patient should be taken to the hospital and then we think of a car as something that can help us get to the hospital quickly and this is why I took a picture of it.” -* Photovoice participant with related photo in Fig. 6.

#### Roads - There is a lack of reliable uncongested roads with priority for emergency vehicles

This barrier was commonly discussed. The potentially unreliable condition of roads was highlighted with a particular emphasis on roads being vulnerable to deterioration in rainy season.

**Fig. 7:**
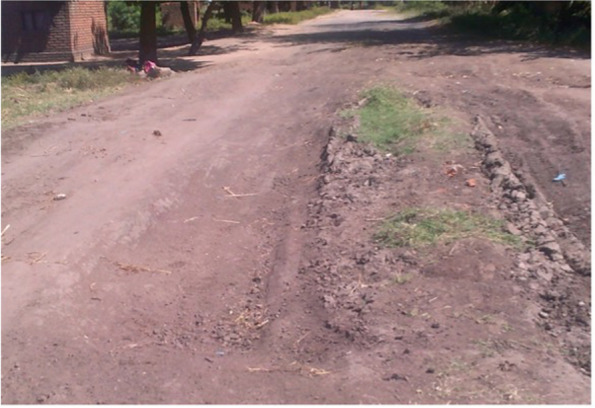
Msewu uheni (bad road)

*“I took a picture of a road; this road is very bad. Where I am staying*,* people have problems of getting to the hospital. When one gets injured at night*,* especially after 8 o’clock*,* we use motorcycles and sometimes someone who is injured cannot sit alone on a motorcycle*,* he needs someone else and that means the fare doubles and it becomes very expensive. All this is because of the condition of the road. Sometimes*,* the owners of the motorcycles refuse to take two people just because the road is bad*,* and we have problems in transporting the sick person to the hospital.” -* Photovoice participant with related photo in Fig. 7.

*“Especially during the rainy season*,* the roads can be viewed … when the rain is falling . no choice but to wait till it stops.” -* Community leader.

*“. we are referring to houses that are built in mountainous areas and because of the rains*,* roads become bad and accessibility difficult and so*,* it becomes difficult for any vehicle to come and pick this patient.” -* Recently injured community member.

**Fig. 8:**
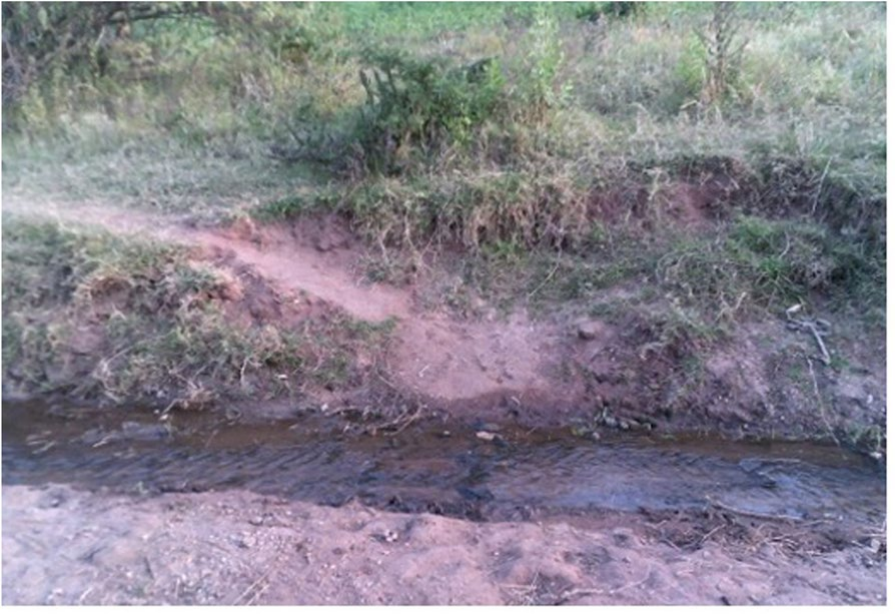
Karavati yawukapo (Culvert washed away)

*“This is a culvert*,* on a stream and this has been carried away by water and people will not be able to carry the patient to the hospital because the culvert has been washed away… you will see that small road which can only be used by people who walk on foot because the oxcart cannot also cross and they can only use a stretcher because of this problem. The road is wide enough*,* but the problem is that the culvert has been washed away and it is not easy to get to the hospital.” -* Photovoice participant with related photo in Fig. 8.

### Delay 3 receiving quality care

#### Physical Resources - There is a lack of reliably available necessary physical resources (e.g. infrastructure, equipment, and consumable materials)

A lack of physical resources were frequently raised across the focus groups and photovoice workshops. Medication availability was emphasised particularly along with other equipment resources including x-ray, scanning and reliable electricity:

*“If medicines are available we can get care quickly” -* Uninjured community member.

*“In addition*,* we can get care quickly at the hospital if all equipment’s are available so that when an injured person is brought*,* they do x-ray*,* scan if all is there*,* the person can get care quickly” -* Uninjured community member.

**Fig. 9:**
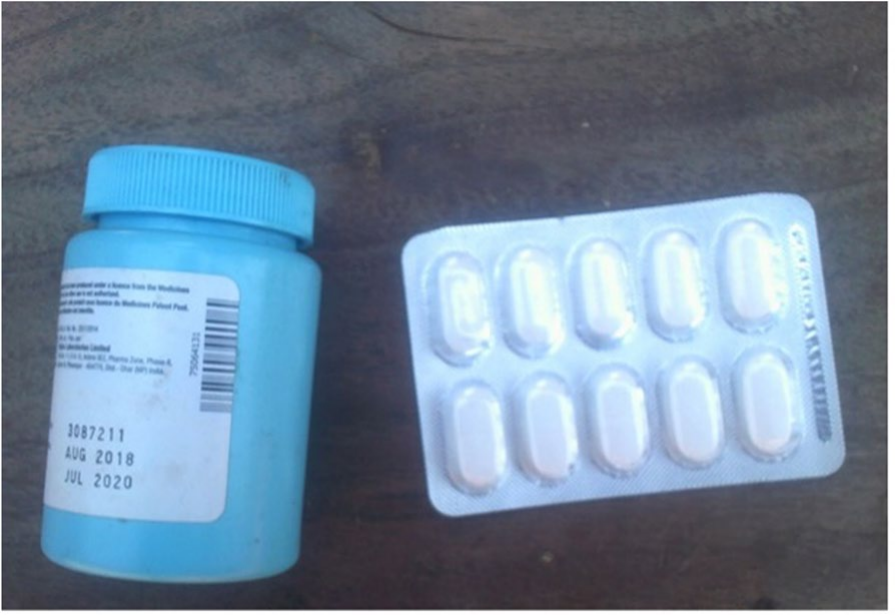
Kuchepa kwa mankhwara (lack of medicines)

*- “I wanted to show that we are already at the hospital and instead of being given medication*,* you are told that there is no medicine and then something is written on your health passport telling you to go and buy for yourself. So*,* I wanted to show lack of medicine at the facility… Sometimes when you get to the hospital you are told*,* oh sorry you have just delayed*,* we are going to give you Bactrim (antibiotic) and you can go and buy the rest.” -* Photovoice participant with related photo in Fig. 9.

#### Payment - difficulties with timely payment for care

Healthcare in government facilities should be free to all nationals within Malawi. Private care and faith-based providers who charge for some services also provide important services, however, financial resource is necessary to make such direct payments for care.

*“What we have been discussing is concerning the government hospitals*,* otherwise*,* for mission hospitals for you to be assisted in good time you ought to have manifest soul*,* can say availability of money.” -* Recently injured community member.

*“According to him*,* this animal can be used as transport and also as cash and my thinking is that this picture should be placed on all the 3 delays*,* because at times the money can be used to settle medical bills so this is why I am proposing that it should be put on all the three delays.” -* Photovoice participant with related photo in Fig. 4.

Participants reported that patients who were perceived to be wealthy would also receive better care and more promptly.

*“You may find someone with a car having headache is given injections and drip of water*,* but I may be sick for three days*,* vomiting and diarrhoea they will just pass by and say go outside you are making the hospital unhygienic.”* - Uninjured community member.

#### Alleged corruption - need for unauthorised payments or gifts to healthcare staff to receive best available treatment

Closely related to difficulty with payments, although not derived from the Delphi study, this was reported by injured and uninjured community members and captured within two photovoice images. Participants reported a requirement to provide additional payments to receive care promptly.

*“Yes*,* if you have money because I have seen some people who are injured instead of treating them*,* they are left aside not attending to them but if you put something what they want inside health passport they will treat you quickly.” -* Uninjured community member.

**Fig. 10:**
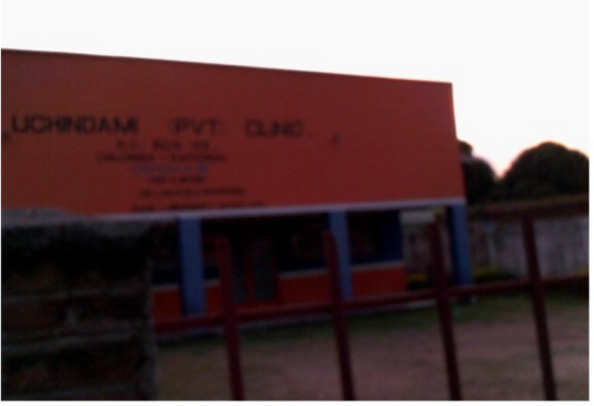
Vimbundi p.a. chipatala (alleged hospital corruption) – (this photo shows the entrance to a clinic)

*“Secondly*,* I get to the hospital and I find the doctors alright*,* but instead of giving me the required care they will just look at me expecting me to give them a little something before they can start helping me. Those of us that come from our homes we may not know but you notice your patient who came about 3 days ago is not getting any assistance and people start telling you that the doctors are expecting something from you before they can start working on your patient. When you meet this doctor privately and give him something he will immediately start working on your patient.”* Photovoice participant with related photo in Fig. 10.

However, a cause and effect relationship between such payments and a better experience of care was questioned by one participant.

*“For example*,* when I got injured*,* I went to the hospital and there was a certain lady who was also injured in the same accident with me and she was just on the bed just next to mine. She used to give them money*,* but they never tended to her for almost a week when she was operated on. The doctors did not remove the glass which was inside her leg and so her leg started getting bad but still she was not attended to. In my case I was there for just a day and I got all the care that is required but I never paid any penny*,* so I think it is just luck. So*,* in the government hospitals money is not really the issue but it depends on how lucky one is.” -* Recently injured community member.

Preferential treatment could also be secured through reputation, by virtue of being a “well known” individual. Although not explicitly personal gain through invested authority, this represents inequitable treatment based on an individual’s influence, separate to, but perhaps associated with, wealth.

*“If you are well-known to the doctors*,* you can get care quickly.” -* Uninjured community member -.

Regarding participant reported experience of conducting photovoice, challenges related to permissions to take photographs of people or buildings were raised. Participants reported being clear, following the training. of the need to ensure individuals provide consent to be photographed. Participants reported that some people, despite thorough explanation, were not happy to be photographed. Two participants reported additional ideas for photos that they did not manage to capture. One participant had an idea to capture a home-made stretcher, another wanted to capture a traditional healer, but neither had time within the study’s confines. Overall, the experience was reported as being positive, helping participants to see some challenges within the community. However, the participants did not report being empowered to address these barriers themselves but preferred external bodies, governmental and non-governmental organisations to aid through projects.

## Discussion

Our combined FGD and photovoice study was a novel application to study an injury care system through a Three Delays framework. The method aimed to encourage participants to use visual images to tell a story, in this case of a barrier or problem, as they perceive it [31]. We attempted to use photographs to express a complex set of concepts and information that might not be fully possible through verbal discussion alone [62]. Delay 1 was the most frequently captured delay by photovoice images whilst the individual barriers most frequently described were “*transport*” and “*roads*”.

That Delay 1 was the most frequently captured delay by photovoice images likely reflects the high priority of this delay for those recently injured community-based participants. Although some authors have found little delay in seeking care following injury [63], several other studies have found quantitative or qualitative evidence. Delays to seeking care represented two fifths of reasons for delays to fracture care across 18 different LMICs [64]. Low levels of care seeking following injury have been described in similar LMIC settings [65–67], more pronounced for rural populations than urban in a study from Ghana [68]. However, some preference for Delay 1 images we observed could be due to participants taking photos of subjects easy to take rather than necessarily more important. Proximity to community issues and the relative accessibility to photograph them might have emphasised these more than secondary care issues due to difficulties in visiting these locations. This might explain why the physical distance from the place of injury to an appropriate healthcare facility (“*distance*”) was not captured within a photovoice image.

The individual barriers most frequently described were “*transport*” and “*roads*”. A wide range of transport issues were discussed; however, formal prehospital emergency transport was rarely mentioned specifically. There is no formal Emergency Medical System (EMS) in place in Malawi, and access to professional prehospital care in Malawi is *“almost non-existent”.* [69] This observation is prevalent across sub-Saharan Africa as almost all of Africa’s population cannot access EMS [70]. Informal methods, ranging from motorised transport in cars and motorbikes to oxcarts were evidenced in our study. Informal methods for urgent transport are common in similar settings [30, 64, 71–74]. Both securing and paying for this informal transport remain important barriers to reaching trauma care [28, 29, 75, 76]. 

With respect to the barrier “roads” this was discussed primarily in relation to the reliability of the road condition, rather than congestion or emergency vehicles priority access. Similar findings have been shown in other countries [29, 77]. Seasonal variation, particularly in relation to rainy season, can adversely impact actual travel times, especially on non-paved roads [78]. This came across clearly as relevant in our assessment through FGDs and illustrative photovoice images. This finding chimes with those from a facility process mapping study within the same health system [79]. Factoring in the impact of seasonally variable road conditions is important to incorporate in attempts to measure and improve accessibility of care following injury [80]. 

The photovoice study did not describe any additional barriers not covered in the FGDs. Since the photovoice study was a follow on extension from a FGD on the same issue [81], this may have influenced photo selection to reflect barriers already discussed within the FGD process and explain some of the convergence and divergence of findings seen. This sequence of study conduct allowed time and detailed discussion to grasp the Three Delays framework concept and consider important barriers in the health system. However, it may have inadvertently restricted innovative thinking and novel barrier generation through the use of photovoice. Stifling creativity in photovoice should ideally be avoided [55, 82]. The lack of photos exploring the barriers “*healthcare literacy*” and “*patient cooperation*” is also noteworthy. Social context could explain why specific photos may or may not have been taken [31]. There may have been reluctance to capture images that made community participants appear illiterate, ignorant or naïve (healthcare literacy), or similarly uncooperative or unhelpful (patient cooperation). However, some images taken did have negative connotations. Participants could also have taken photos based on what they thought we as researchers had wanted to see, having discussed the issues in the earlier FGD [38]. However, we also found that participants interpreted photovoice images as encapsulating several interrelated barriers often cutting across multiple delays. This was likely encouraged using a Venn diagram discussion stimulus. It illustrates that participants engaged with the study had grasped both the Three Delays framework and method of photovoice.

Our study used health system barriers from a prior Delphi study as a starting framework for deductive and subsequent inductive analysis [17]. This prior study intended to identify barriers important to evaluate when assessing health system preparedness for injury care and we recognise that the presence or absence of barriers can vary between countries, contexts and health systems [83]. We found no evidence of the Delphi described barriers “*prehospital care”*,* “communication”* and “*coordination”* in our qualitative community study. The lack of focus on these Delay 2 barriers relating to a mature functioning formal prehospital emergency care system could have been due to the local context lacking formal EMS [69]. Participants cannot desire something they are unaware of during unprompted discussions. Educating community members about features of more mature emergency health systems may have some value [29]. However, this finding also supports using additional data sources and methodological approaches additional to community-based focus groups when prioritising health system strengthening interventions [8]. 

Although a purpose of photovoice is to use photographs to generate discussion, rather than for the photos to completely match the concept that they are being used to illustrate, we observed a relative lack of taking and describing photos in the abstract to convey a conceptual barrier to care. This perhaps explains the lack of photovoice evidence for the barriers related to perceptions of care accessibility, fear or lacking courage, and healthcare literacy. Photos tended to directly illustrate an issue. In other photovoice studies, more creative use of photos and abstract interpretations have been described. For example, a photo showing the complicated intertwining of tree branches was used in a study of veteran healthcare to illustrate the perceived impenetrable complexity of the care system experienced [84]. Although we encouraged creativity during participant training, this creative scope could have been better illustrated by providing such examples. However, we were keen not to put specific ideas for photos into our participants’ minds, but rather encourage their generation [85]. 

We saw some overlap of images captured. Several participants, for example, chose to take very similar photos of notes of Malawian currency (kwacha). One possible explanation is that participants could have discussed this idea together rather than showing independent thought. Alternatively, it could have been the most obvious way to convey the problem financial issues play in accessing quality injury care, which is undoubtedly evidenced across this study. Greater creativity might also be linked to greater literacy levels [86, 87]. 

Taking photos of identifiable individuals in photovoice studies poses an ethical challenge. How best to manage such challenge is debated by photovoice researchers [88]. We spent time devising and explaining a consent taking procedure for the use of images involving people. In the event, few photos contained identifiable people. We quickly reconfirmed consent as these individuals were often found close to the participant’s home, an experience not always replicated [88]. Our alternative strategy, blurring identifiable individuals’ faces if consent was not obtainable, was not required. Such an approach would allow broader use of photograph images, including perhaps of crowds, in future. The requirement to obtain written consent may have discouraged taking photos of people, leading some researchers to advocate verbal consent being sufficient for such studies [88]. Written permission of those individuals photographed was deemed ideal, but not practical, in other Malawian contexts [32]. 

There are some other limitations to our study. We only included participants able-bodied enough to take photographs. People with an injury-related disability might have been excluded, and their experiences not captured. We could have allowed family members to take photos under such participant direction as trialed elsewhere [38], although this was considered too complicated in our study. The photovoice participants had all received healthcare for their injury, alternative barriers may have been emphasised if the participants had not accessed care. We used photovoice in a community setting from where it originates [31], . Healthcare workers may have chosen different images with different meaning to emphasise. To encourage a breadth of photo subjects, perhaps especially from care settings, we could have provided more time for participants with the camera and offered support in capturing other images such as through offering transportation. However, this would have increased both the time and cost implications for the study and added additional ethical complexity.

When we asked about additional photos, the main issue mentioned was the time to photograph a traditional healer, but support with transport was not requested. Photovoice studies can vary substantially in the time allotted to take photos which is likely to be driven by researcher time scales and budget [55]. Whilst some comparable studies in Malawi have used more extended time periods for photographing subjects [35], photo-taking windows of only a few days is common [32–34]. Within the injured community member focus group and photovoice individuals there was a balance of sex and broad range of ages. A similar balance was seen with the community leader participants although the uninjured community members were predominantly female and younger on average. Whilst this may have enabled discussion on sex-based issues, it was actually community leaders who identified the cultural norm of male household heads making care seeking decisions as a barrier to care.

## Conclusion

We used a novel application of focus group discussions with photovoice technique to assess barriers for seeking, reaching, and receiving injury care in Northern Malawi. We were able to identify several barriers active within this health system and allow participants to illustrate how some barriers impact on more than one phase of delay. The method was quick and low cost to perform with participants able to grasp the technique and research question effectively. We advocate use of this approach to complement similar future health system assessments.

## Data Availability

Availability of data and materialsData can be made available from Malawi Epidemiology and Intervention Research Unit (MEIRU) on reasonable request and following the signing of a data transfer agreement. Data enquiries should be sent to info@meiru.mw and should indicate the title of the paper.

## Abbreviations

EMS: Emergency Medical System
FGD: Focus Group Discussion
HDSS: Health and Demographic Surveillance Site
MEIRU: Malawi Epidemiology and Intervention Research Unit
CHAM: Christian Health Association of Malawi
LIC: Low-income Country
HIC: High-income Country
LMIC: Low– and Middle- Income Country
DALYs: Disability Adjusted Life Years
